# The role of the epidermal growth factor receptor tyrosine kinase inhibitors as therapy for advanced, metastatic, and recurrent non-small-cell lung cancer: a Canadian national consensus statement

**DOI:** 10.3747/co.v16i1.393

**Published:** 2009-01

**Authors:** P.M. Ellis, W. Morzycki, B. Melosky, C. Butts, V. Hirsh, F. Krasnoshtein, N. Murray, F.A. Shepherd, D. Soulieres, M.S. Tsao, G. Goss

**Affiliations:** * Juravinski Cancer Centre, Hamilton, ON; † QEII Health Sciences Centre, Nova Scotia Cancer Centre, Halifax, NS; ‡ BC Cancer Agency, Vancouver, BC; § Cross Cancer Institute, Edmonton, AB; || Royal Victoria Hospital, Montreal, QC; # MedWrite, Montreal, QC; ** Princess Margaret Hospital, Toronto, ON; †† Centre Hospitalier de l’Université de Montréal, Montreal, QC; ‡‡ The Ottawa Hospital Cancer Centre, Ottawa, ON

**Keywords:** Non-small-cell lung cancer, targeted therapy, epidermal growth factor receptor, tyrosine kinase inhibitor, molecular marker

## Abstract

**Purpose:**

To provide consensus recommendations on the use of epidermal growth factor receptor tyrosine kinase inhibitors (egfr-tkis) in patients with advanced or meta-static non-small-cell lung cancer (nsclc).

**Methods:**

Using a systematic literature search, phase ii trials, randomized phase iii trials, and meta-analyses were identified for inclusion.

**Results:**

A total of forty-six trials were included. Clear evidence is available that egfr-tkis should not be administered concurrently with platinum-based chemotherapy as first-line therapy in advanced or metastatic nsclc. Evidence is currently insufficient to recommend single-agent egfr-tkis as first-line therapy either in unselected populations or in populations selected on the basis of molecular or clinical characteristics. Following failure of platinum-based chemotherapy, the evidence suggests that second-line egfr-tkis or second-line chemotherapy result in similar survival. Quality of life and symptom improvement for patients treated with an egfr-tki appear better than they do for patients treated with second-line docetaxel. Sequence of therapy may not appear to be important, but if survival is the outcome of interest, the goal should be to optimize the number of patients receiving three lines of therapy. Based on available data, molecular markers and clinical characteristics do not appear to be predictive of a differential survival benefit from an egfr-tki and therefore those factors should not be used to select patients for egfr-tki therapy.

**Conclusions:**

The egfr-tkis represent an additional therapy in the treatment of advanced or metastatic nsclc. The results of ongoing clinical trials may define the optimal role for these agents and the effectiveness of combinations of these agents with other targeted agents.

## 1. INTRODUCTION

Lung cancer represents a major health burden in Canada. Approximately 23,300 new lung cancer cases and 19,900 deaths from lung cancer occurred in 2007, most of which were non-small-cell lung cancer (nsclc)^1^. Most of these patients either present with or develop metastatic disease at some point during their illness; potentially, they are candidates for systemic therapy approaches such as chemotherapy.

Until the late 1990s, therapeutic nihilism about the benefit of systemic chemotherapy in the treatment of advanced and metastatic nsclc was widespread. Publication of the Non-small Cell Lung Cancer Collaborative Group meta-analysis in 1995 established the association of first-line platinum-based chemotherapy with a modest improvement in survival for patients with metastatic disease^2^. The introduction of newer drugs such as vinorelbine, gemcitabine, paclitaxel, and docetaxel have resulted in further small improvements, although most patients still experience disease progression within a short time, with a median time to progression (ttp) of approximately 4 months 3–5.

At the time of progression following platinum-based chemotherapy, many patients maintain a good performance status (ps) and may be candidates for further systemic therapy. Recent trials have established that second-line chemotherapy with docetaxel^6^–^9^ improves survival and quality of life (qol) as compared with best supportive care (bsc) and that survival of patients treated with docetaxel or pemetrexed is similar^10^. Guidelines for the management of nsclc, including those from Cancer Care Ontario’s Program in Evidence-Based Care (cco-pebc)^11^ now recommend either of those agents as second-line chemotherapy options^11^,^12^.

Despite these advancements in the treatment of nsclc, there is still a strong need for additional and better treatment options. Recently, a greater understanding of the molecular abnormalities associated with nsclc has led to evaluation of new therapeutic targets for nsclc. The epidermal growth factor receptor (egfr) is one target commonly overexpressed in nsclc^13^–^15^. Early-phase clinical trials showed that egfr tyrosine kinase inhibitors (tkis) such as erlotinib and gefitinib had antitumour activity, and this finding prompted their further evaluation in advanced nsclc^16^ These agents have been evaluated extensively in phase ii and iii trials over the last few years, confirming the promising activity seen in phase i trials, and the tkis have been incorporated into treatment algorithms for patients after progression on standard chemotherapy options^11^.

Because of a favourable toxicity profile of the tkis, many clinicians felt that it might be appropriate to expand their role in the treatment of advanced and metastatic nsclc. A need therefore exists to clarify the role of egfr-tkis in the treatment of nsclc. The present paper represents a consensus view of a representative sample of Canadian lung cancer medical oncologists on the role of egfr-tkis in the treatment of nsclc based on a systematic review of currently available evidence.

## 2. MATERIALS AND METHODS

Medical oncologists specializing in thoracic oncology from five provinces across Canada were invited to participate in a consensus meeting. Six oncologists attended the consensus meeting, and three additional oncologists, plus one pathologist, provided input into the consensus process. Three key questions were identified to be addressed by the group:

- What is the role of egfr-tkis as first-line therapy of advanced or metastatic nsclc as a single agent or in combination with chemotherapy?
- What is the role of egfr-tkis following progression after platinum-based chemotherapy (single-agent egfr-tki vs. bsc, egfr-tki vs. chemotherapy, and egfr-tki in combination with another agent)?
- Do any patient subpopulations, or clinical and molecular characteristics, predict for additional benefit from egfr-tki therapy?

### 2.1 Literature Search

A search of the medline database for 2000–2007 was conducted using the terms “non-small-cell lung cancer,” “epidermal growth factor receptor tyrosine kinase inhibitor,” “erlotinib,” and “gefitinib.” The search excluded articles prior to 2000, because the egfr-tkis are new agents and their initial phase i trials were known to be conducted during the selected time period. Conference proceedings of the American Society of Clinical Oncology 2000–2007 and the International Association for the Study of Lung Cancer 2007 World Conference on Lung Cancer were also searched. Finally, the list of included articles was reviewed by the consensus panel for omissions.

### 2.2 Study Selection Criteria

Articles published as full reports or as abstracts and conference presentations were included if they focused on

- egfr-tki alone or in combination with chemotherapy in the first-line setting,
- egfr-tki as second- or third-line therapy following progression of platinum-based chemotherapy, or
- clinical and molecular characteristics that may predict additional benefit from egfr-tki therapy.

The literature search results were reviewed by two authors (PE, FK), and articles that met the foregoing criteria were selected for retrieval. The outcomes of interest were overall survival (os), time to disease progression, tumour response rate, molecular and clinical predictors of benefit from egfr-tki therapy, and qol or symptom improvement. Single-arm phase ii trials were included only if no data from randomized trials were available. Forty-three individual trials (eight phase iii, eleven randomized phase ii, and twenty-four single-agent phase ii trials) met the eligibility criteria for the present consensus statement. Only studies published in English were considered.

### 2.3 External Review

Final consensus statement draft recommendations were distributed electronically to reviewers. The review panel consisted of practitioners who had attended the consensus meeting and others who were not in attendance. The comments resulting from this review were incorporated into the final document.

## 3. RECOMMENDATIONS AND KEY EVIDENCE

### 3.1 First-Line Treatment

What is the role of egfr-tkisas first-line therapy of advanced or metastatic nsclc as a single agent or in combination with chemotherapy?

#### 3.1.1 What Is the Role of Single-Agent EGFR-TKIs in Chemonaïve Patients with NSCLC?

##### Key Evidence

Fourteen single-arm phase ii trials (*n* = 1026) and one randomized phase ii trial (*n* = 201) evaluated single-agent erlotinib 150 mg or gefitinib 250 mg daily as first-line therapy of stage iiib/iv nsclc (TABLE I). In general, patients had an Eastern Cooperative Oncology Group ps of 0–2 and were not selected for clinical or molecular characteristics reported to be associated with improved response to an egfr-tki. Substantial variability was observed in the response rate to single-agent egfr-tkis (range: 4%–55%, with an additional 20%–46% achieving disease stabilization). The time to disease progression ranged from 1 month to 6.6 months, with median survival varying between 2.9 months and 14.1 months, and 1-year survival being 24%–58.2% 17–22,24,26,27,30–36,38,39.

A single randomized placebo-controlled trial compared gefitinib to bsc in patients with poor performance (ps 2–3) unsuitable for chemotherapy. The observed response rate was only 6%, and the trial failed to demonstrate significant improvement in either TTP or OS^33^.

Among the trials in unselected populations, qol and symptom improvement data were inconclusive 17–22,24,26,27,30–36,38,39. In the single randomized trial, the proportion of patients reporting qol and symptom improvement appeared similar for gefitinib and bsc (21.1% vs. 20.0% and 28.3% vs. 23.3% respectively)^33^. Several other authors also reported no significant improvement in qol over time 24,31. However, Spigel reported improvement or no change in qol [using the Functional Assessment of Cancer Therapy–Lung (fact-l)] in 82% of patients, and improvement or control in lung cancer symptom (lcs) response in 48% of patients^19^. Pérez–Soler reported significant improvements in pain scores at 2 weeks and improvement in emotional functioning during the first 4 weeks of therapy^17^ (TABLE I). In general, these qol analyses involved small numbers of patients in the absence of control groups and should be interpreted cautiously.

The remaining five phase ii trials selected patients based on the presence of activating mutations of the *EGFR* gene (*n* = 85) or of clinical characteristics associated with high response rate to treatment (*n* = 40). The trials included patients with stage iii or iv nsclc and ps 0–2, and evaluated either erlotinib 150 mg or gefitinib 250 mg daily. Higher response rates were observed in these selected populations (range: 30%–90%) as compared with the unselected populations described earlier^23^,^25^,^28^,^29^,^37^,^40^. Longer time to disease progression was also observed (5.6–13.3 months). Median survival was 15.4 months in one trial^40^ and was either not reported or not reached in the others 23,25,28,29,37. This activity appears encouraging, but randomized trials comparing egfr-tki therapy to chemotherapy are needed to draw firm conclusions.

#### Consensus Recommendation

The evidence is currently insufficient to recommend first-line single-agent egfr-tki therapy in the treatment of advanced or metastatic nsclc. These recommendations apply both to unselected populations and to patients selected on the basis of activating mutations of the *EGFR* gene or of clinical characteristics predictive of higher response to therapy.

There is evidence of tumour response to single-agent egfr-tki as first-line therapy for advanced nsclc. Response rates to egfr-tki therapy appear to be higher in patients selected on the basis of activating mutations of the *EGFR* gene.

Randomized trials are needed to evaluate the effect of first-line egfr-tki on survival.

#### 3.1.2 What Is the Role of Single-Agent EGFR-TKIs in Patients with Adenocarcinoma with Bronchioloalveolar Features?

##### Key Evidence

The literature search identified a consensus document on systemic therapy of bronchi oloalveolar carcinoma (bac)^41^. It states that there is no evidence to confirm or refute the assertion that the sensitivity of bac to chemotherapy is any different from that of other histologic subtypes of nsclc.

Three phase ii trials in ps 0–2 patients with stage iii/iv bac (*n* = 326) evaluated either erlotinib 150 mg or gefitinib 250 mg daily (TABLE II). Patients were predominantly chemotherapy-naïve. Response rates ranged from 9% to 21%, with disease stabilization in an additional 16%–36%. The survival data demonstrated time to disease progression of between 3.0 months and 3.7 months, and median survival of 13.0–17.1 months^42^–^45^. In one study, shorter progression-free survival (pfs) and os were independently associated with non-mucinous as compared with mu-cinous bac (pfs: 2.6 months vs. 11.3 months, *p*=0.002; os: 10.7 months vs. not reached, *p* = 0.003)^44^,^45^.

##### Consensus Recommendation

There is no evidence to suggest that bac should be treated differently from other types of nsclc. The evidence is currently insufficient to recommend egfr-tkis as first-line therapy for the treatment of bac.

#### 3.1.3 What Is the Role of First-Line EGFR-TKIs in Combination with Platinum-based Chemotherapy in Patients with NSCLC?

##### Key Evidence

Four large randomized trials evaluated egfr-tkis in combination with platinum-based chemotherapy in patients with good ps with stage iii/iv nsclc (*n* = 4348, TABLE III). Patients were treated with either gemcitabine and cisplatin [gemcitabine 1250 mg/m^2^ intravenously (IV) on days 1 and 8, and cisplatin 80 mg/m^2^ IV on day 1 of a 21-day cycle] or carboplatin and paclitaxel [carboplatin area under the curve (auc) 6 IV on day 1, and paclitaxel 200 mg/m^2^ IV on day 1 of a 21-day cycle] with or without erlotinib 150 mg or gefitinib 250 mg or 500 mg daily. Response rates varied between the trials; however, all four trials failed to demonstrate any improvement in response rate with the addition of an egfr-tki to platinum-based chemotherapy^46^–^49^. Time to worsening of symptoms did not differ significantly between the groups^46^,^47^,^49^.

No differences were observed in time to disease progression or in median and 1-year survival between patients randomized to chemotherapy alone and those randomized to chemotherapy plus an egfr-tki (see TABLE III).^46^–^49^

##### Consensus Recommendation

Clear evidence from four randomized trials shows that concurrent administration of an egfr-tki with first-line platinum-based chemotherapy does not prolong survival in unselected patients with nsclc.

#### 3.1.4 What Is the Role of Single-Agent EGFR-TKIs Compared with Chemotherapy in Chemonaïve Patients with NSCLC?

##### Key Evidence

Two randomized trials compared first-line therapy with an egfr-tki with chemotherapy in chemonaïve patients with stage iii/iv nsclc and ps 0–2 (*n* = 299, TABLE IV) 50,52. Lilenbaum randomized patients with poor ps (score of 2) to treatment with either carboplatin and paclitaxel (carboplatin auc 6 and paclitaxel 200 mg/m^2^ for 4 cycles) or erlotinib 150 mg daily 52; Crinò randomized elderly patients (more than 70 years of age) to vinorelbine 30 mg/m^2^ IV on days 1 and 8 of a 21-day cycle or gefitinib 250 mg daily^50^.

Lilenbaum observed a higher response rate among patients treated with chemotherapy than with erlotinib [overall response (or): 12% vs. 2%; or + stable disease (sd): 53% vs. 39%]. Additionally, patients randomized to carboplatin–paclitaxel had a longer time to progression (3.5 months vs. 1.9 months) and a greater survival (9.1 months vs. 6.6 months), although these differences were not statistically significant^52^.

Crinò observed similar activity from vinorelbine and gefitinib (or: 5.1% vs. 3.1%; or+sd: 53% vs. 43%). The pfs favoured vinorelbine, but this difference was not statistically significant [hazard ratio (hr): 1.19; 95% confidence interval (ci): 0.85 to 1.65]. No difference in overall survival was observed (hr: 0.98; 95%ci: 0.66 to 1.47). The groups showed no difference in overall qol (by fact-l) and in lcs. Gefitinib appeared to be better tolerated than vinorelbine^50^.

A third trial evaluated various doses and schedules of erlotinib with carboplatin and paclitaxel^51^. No significant differences were observed among the three treatment groups (TABLE IV).

##### Consensus Recommendation

The evidence is currently insufficient to recommend the use of an egfr-tki over chemotherapy in the first-line therapy of patients with nsclc. Available evidence raises the possibility that survival of patients with poor ps treated with first-line egfr-tki may be less than that of patients treated with platinum-based chemotherapy.

### 3.2 Second-Line and Subsequent Treatment for Relapsed or Recurrent Disease

What is the role of egfr-tkis following progression after platinum-based chemotherapy (single-agent egfr-tki vs. bsc, egfr-tki vs. chemotherapy, and egfr-tki in combination with another agent)?

#### 3.2.1 What Is the Role of EGFR-TKIs as Second- or Third-Line Therapy Following Progression of Platinum-based Chemotherapy?

##### Key Evidence

Two guidelines developed by cco-pebc, addressing the role of an egfr-tki as subsequent therapy for nsclc, were identified 11,53. Both documents recommend the use of erlotinib as second- or third-line therapy for nsclc in patients who are not candidates for further chemotherapy.

Four randomized phase ii and iii trials in ps 0–2 patients with stage iii/iv nsclc who were not considered candidates for further chemotherapy examined egfr-tkis as subsequent therapy following progression of platinum-based chemotherapy (*n* = 2849, TABLE V). Two large phase iii studies evaluated erlotinib 150 mg (br.21) or gefitinib 250 mg [isel (Iressa Survival Evaluation in Lung Cancer)] daily compared with placebo 56,57, and two randomized phase ii studies [ideal 1 and 2 (Iressa Dose Evaluation in Advanced Lung Cancer 1 and 2)] compared two doses of gefitinib (250 or 500 mg daily)^54^,^55^. In the ideal 1 and 2 trials, no differences were observed in any outcomes examined between gefitinib 250 mg and 500 mg daily.

Results of the br.21 and isel trials demonstrated that erlotinib (2.2 months vs. 1.8 months) and gefitinib (3.0 months vs. 2.6 months) significantly prolong time to disease progression 56,57. Statistically significant improvements were also seen in os with erlotinib as compared with placebo (6.7 months vs. 4.7 months, *p* < 0.001) 56, and a trend toward improved survival was observed with gefitinib (5.6 months vs. 5.1 months, *p* = 0.087) 57.

In the br.21 trial, patients receiving erlotinib experienced significantly longer time to deterioration in several lung cancer-related symptoms (cough, pain, dyspnea) and in overall physical function 58. In the isel trial, a greater proportion of patients randomized to gefitinib experienced improvement in disease-related symptoms (27% vs. 22%). Similarly, patients randomized to gefitinib experienced a significantly greater improvement in lcs scores (−1.38 vs. −0.86, *p* = 0.019)^57^.

##### Consensus Recommendation

In patients with advanced or metastatic nsclc who are not candidates for further chemotherapy, the use of an egfr-tki (as compared with placebo) can result in improved survival. The use of an egfr-tki in patients with nsclc who are not candidates for further chemotherapy can result in significant improvements in disease-related symptoms, and as compared with bsc alone, can delay time to symptom progression.

#### 3.2.2 What Is the Role of EGFR-TKIs Compared with Chemotherapy Following Progression of Platinum-based Chemotherapy?

##### Key Evidence

Seven randomized phase ii and iii trials examined an egfr-tki as compared with chemotherapy following progression of platinum-based chemotherapy in patients with stage iii/iv nsclc and ps 0–2 (*n* = 2482, TABLE VI).

One randomized phase ii trial^59^ and two randomized iii trials phase 62,65,66 evaluated gefitinib 250 mg daily vs. docetaxel 60 or 75 mg/m^2^ IV every 3 weeks (*n* = 2096). The response rate with gefitinib was significantly higher than that with docetaxel in a Japanese population (22.5% vs. 12.8%, *p* = 0.009)^65^,^66^. However no differences were observed in response rate between gefitinib and docetaxel in the other two trials^59^,^62^. No significant differences were observed in ttp or os in patients treated with gefitinib or docetaxel. In the trial by Niho *et al.,* the proportion of patients randomized to docetaxel who received third-line egfr-tki therapy was greater than the proportion of patients randomized to gefitinib who received third-line chemotherapy. That trial did not meet its primary outcome of non-inferiority of gefitinib (upper limit of 95% ci ≤ 1.25) as compared with docetaxel (hr: 1.12; 95% ci: 0.89 to 1.40)^65^,^66^. However, the larger interest trial (Iressa non-small-cell lung cancer trial evaluating response and survival against Taxotere) demonstrates that gefitinib was non-inferior to docetaxel (hr: 1.02; 95% ci: 0.905 to 1.15), in which the definition of non-inferiority accepted a ci going up to 1.154^62^. The proportion of patients receiving effective third-line therapy was similar between the two treatment arms in that trial.

Another four randomized phase ii studies evaluated gefitinib 250 mg or erlotinib 150 mg daily with other agents (oral vandetanib 300 mg daily^60^; bortezomib 1.6 mg/m^2^ IV on days 1 and 8 of a 21-day cycle^64^; vinorelbine 15 mg/m^2^ IV on day 1, and gefitinib 250 mg daily on days 2–14 every 2 weeks^61^; bevacizumab 15 mg IV on day 1 every 3weeks; docetaxel 75 mg/m^2^ on day 1 of a 3-week cycle; pemetrexed 500 mg/m^2^ on day 1 of a 3-week cycle)^63^ either as single agents or in combination (*n* = 386, TABLE VI). No firm conclusions can be drawn from any of these trials, although compared with erlotinib alone, the combination of erlotinib plus bevacizumab demonstrated improvement in response rate (17.9% vs. 12.2%), ttp (4.4 months vs. 3.0 months), and os (13.7 months vs. 8.6 months)^63^. A phase iii trial of that combination is ongoing. Fully powered phase iii trials are ongoing to compare gefitinib with vandetanib and to assess whether bevacizumab adds to the efficacy of single-agent erlotinib.

##### Consensus Recommendation

The evidence suggests that second-line egfr-tki or second-line chemotherapy results in similar survival. Sequence does not appear to be important, but if survival is the outcome of interest, the goal should be to optimize the number of patients receiving three lines of effective therapy. The evidence is currently insufficient to recommend second-line therapy with a combination of an egfr-tki and another targeted agent. Ongoing randomized phase iii trials are currently addressing these questions.

#### 3.2.3 How Do QOL and Symptom Control Compare in Patients Treated with Chemotherapy as Compared with EGFR-TKIs?

##### Key Evidence

Two of the three trials that compared gefitinib and docetaxel also examined qol and symptom improvement^59^,^62^.

In the sign trial (Second-Line Indication of Gefitinib in nsclc), a greater proportion of patients randomized to gefitinib experienced symptom improvement as assessed by lcs (36.8% vs. 26%) and qol improvement as assessed by the fact-l (33.8% vs. 26%)^59^. In addition, in the interest trial, significantly more patients randomized to the gefitinib arm showed improvements in fact-l score (25.1% vs. 14.7%, *p* < 0.0001) and trial outcome index (17.3% vs. 10.3%, *p* = 0.0026). Symptom improvement rates were also better with gefitinib than with docetaxel, but this difference was not statistically significant^62^.

##### Key Recommendation

Symptom control and qol appear to be better in patients treated with an egfr-tki than in those treated with either bsc or second-line chemotherapy with docetaxel. In decisions about treatment following failure of platinum-based chemotherapy, qol and patient choice are important.

#### 3.2.4 What Is the Role of Single-Agent EGFR-TKI Therapy in Previously Treated Patients with EGFR Gene Mutations or High Gene Copy, or EGFR Protein Expression?

##### Key Evidence

Four single-arm phase ii trials evaluated gefitinib 250 mg daily in patient populations (n = 117) selected for the presence of activating mutations of the *EGFR* gene assessed by polymerase chain reaction (pcr) analysis or for high *EGFR* gene copy assessed using fluorescence *in situ* hybridization (fish). Patients had stage iii/iv disease and ps 0–2, and most had received prior chemotherapy. High response rates were observed (48%–90%)^67^–^70^. Time to disease progression ranged from 6.4 months to 12.9 months, with a median survival of 15.5 months reported in one study^69^. Given that *EGFR* mutations are thought to represent a favourable prognostic factor, the significance of these data are unclear, and randomized trials are needed to determine if the survival of patients with *EGFR* mutations or high *EGFR* gene copy treated with an egfr-tki is superior to that of similar patients treated with second-line chemotherapy.

##### Consensus Recommendations

There is evidence that patients with previously treated nsclc and *EGFR* mutations or increased *EGFR* gene copy respond to an egfr-tki. However, the evidence is insufficient at this time to select patients for egfr-tki therapy rather than for second-line chemotherapy based on any *EGFR* marker.

### 3.3 Clinical and Molecular Predictors of Benefit

Do any patient subpopulations, or clinical and molecular characteristics, predict for additional benefit from egfr-tki therapy?

#### 3.3.1 What Are the Molecular Characteristics that Predict Additional Benefit from egfr-tki Therapy?

##### Key Evidence

###### Clinical Predictors of Response to an egfr*-*tki

TABLE VII summarizes the trials examining clinical predictors of response. Data are available from the ideal 1, ideal 2, br.21, and isel trials. Analyses from the ideal 1 and 2 trials demonstrated that ad-enocarcinoma (13% vs.4%) and female sex (19% vs. 3%) both significantly predict response to gefitinib^55^. Additional clinical predictors of response were observed in the br.21 trial. In that study, clinical characteristics associated with higher response to erlotinib included adenocarcinoma (13.9% vs. 4.1%, *p* < 0.001), never smokers (24.7% vs. 3.9%, *p* < 0.001), female sex (14.4% vs. 6%, *p* = 0.006), and Asian ethnicity (*n* = 427: 18.9% vs. 7.5%, *p* = 0.002)^56^,^71^–^73^. Consistent with the br.21 results, subset analysis from the isel trial also demonstrated that adenocarcinoma (11.9% vs. 4.8%), never smokers (18.1% vs. 5.3%), female sex (14.7% vs. 5.1%), and Asian ethnicity (12.4% vs. 7.5%) were predictors of response to gefitinib (*n* = 1439)^57^.

###### Clinical Predictors of Survival with an egfr-tki

TABLE VIII summarizes clinical predictors of survival for patients receiving therapy with an egfr-tki 57,71–79. In the br.21 trial, the only clinical characteristic that predicted greater effect on survival for erlotinib as compared with supportive care alone was a history of never having smoked (hr: 0.4 vs. 0.9; *p* = 0.02). There was no evidence of any differential survival effect for histology (hr: 0.7 adenocarcinoma vs. 0.8 non-adenocarcinoma), sex (hr: 0.8 males vs. 0.8 females), or ethnicity (hr: 0.6 Asian vs. 0.8 non-Asian) 71–73,77,78. The isel trial demonstrated significantly improved survival among patients randomized to gefitinib for never smokers (hr: 0.67; 95% ci: 0.49 to 0.92) and for patients of Asian ethnicity (hr: 0.66; 95%ci: 0.48 to 0.91)^57^. There was a trend toward improved survival for patients with adenocarcinoma treated with gefitinib (hr: 0.84; 95% ci: 0.70 to 1.02). In a subset analysis of all Asian patients from the isel trial, significant improvements in survival were seen for patients with adenocarcinoma (hr: 0.66 vs. 0.86), never smokers (hr: 0.37 vs. 0.85), and female sex (hr: 0.46 vs. 0.80)^76^.

No data were available concerning clinical predictors of survival from the intact (Iressa nsclc Trial Assessing Combination Treatment) 1 and 2 trials^80^. In a subset analysis of never smokers (*n* = 113) from the tribute (Tarceva Responses in Conjunction with Paclitaxel and Carboplatin) trial, a significant improvement in survival was observed from the addition of erlotinib (hr: 0.49; 95% ci: 0.28 to 0.85) 81. Similar findings were observed in talent (Tarceva Lung Cancer Investigation Trial). Improved os and pfs were observed for patients receiving erlotinib who had never smoked (hr: 0.39; *p* = 0.25), although this interaction did not achieve statistical significance^74^,^75^.

In contrast, subgroup analyses from the interest trial comparing gefitinib with docetaxel suggest that these clinical variables do not predict a differential benefit for an egfr-tki over chemotherapy. There was no difference in the survival of patients with adenocarcinoma histology, never smokers, Asian ethnicity, and female sex when treated with either gefitinib or docetaxel^79^.

###### Molecular Predictors of Response to an egfr-tki

The predictive value of various molecular abnormalities have been examined in the randomized trials included in the present consensus document. These include mutations of the *EGFR* gene, increased *EGFR* gene copy assessed by fish or *EGFR* amplification assessed by quantitative pcr, egfr expression [by immunohistochemistry (ihc)], and mutations of the *KRAS* gene. TABLE IX summarizes predictors of response.

The presence of an activating mutation of the *EGFR* gene is associated with an increased likelihood of response to single-agent egfr-tki. Analyses of tumour samples from the ideal 1 and 2 trials (*n* = 425) evaluating gefitinib monotherapy demonstrated that patients whose tumour had an *EGFR* mutation had a better or with gefitinib than did patients lacking the mutation (*n* = 79: 46% vs.10%, *p* = 0.005)^80^. In the br.21 (*n* = 177: 15.8% vs. 7.4%, *p* = 0.35) and isel trials (*n* = 215: 37.5% vs. 2.6%), the presence of an *EGFR* mutation was associated with a nonsignificant increase in response rate. In br.21, when only exon 19 deletion and L858R mutations were considered, the difference in response rate as compared with wild-type *EGFR* or other mutations was significant (27% vs. 7%, *p* = 0.035)^85^. The subset analysis of tumour samples from the intact 1 and 2 trials evaluating the addition of gefitinib to standard first-line chemotherapies demonstrated that patients whose tumours had an *EGFR* mutation had a higher response to chemotherapy plus gefitinib than did those without a mutation (*n* = 170: 72% vs. 55%, *p* = 0.2)^80^. Similar findings were observed in the tribute trial for patients with *EGFR* mutations (*n* = 228: 53% vs. 21%, *p* < 0.01)^82^,^84^, but no statistically significant correlation was observed between response rates and mutation status in the talent trial^74^,^75^.

Increased *EGFR* gene copy or *EGFR* amplification also appears to be associated with an increased response rate to single-agent egfr-tki. The ideal 1 and 2 trials demonstrated that *EGFR* amplification was associated with a higher response to gefitinib than was seen with tumours without *EGFR* amplification; however, this difference was not statistically significant (*n* = 90: risk ratio: 29% vs.15%; *p* = 0.319). Patients with an *EGFR* mutation or gene amplification had a significantly improved response rate as compared with patients with neither *EGFR* amplification nor mutation (60% vs. 10%, *p* = 0.0011)^80^. Within the br.21 trial, high *EGFR* gene copy or amplification also was associated with a significantly higher response to erlotinib (*n* = 91: 21% vs. 5%, *p* = 0.02)^71^,^77^,^85^. Similar findings were observed in the isel trial (*n* = 317: 16.4% vs. 3.2%)^83^.

In intact 1 and 2, there were no differences in response with and without *EGFR* amplification (n = 235: 56% vs. 53%, *p* > 0.05)^80^. Interestingly, analysis of tumour samples from the tribute study demonstrated a lower response rate among patients whose tumours demonstrated *EGFR* amplification^82^,^84^. It is important to note that fish was used to assess *EGFR* gene copy status in the br.21, isel, and tribute studies^82^–^85^, but that quantitative pcr was used in the ideal and intact studies^80^. High *EGFR* gene copy by fish includes cases of *EGFR* high polysomy and of *EGFR* amplification alike^82^–^85^, but quantitative pcr results include cases of *EGFR* gene amplification only^80^. Thus, the two results are not entirely comparable.

Fewer data are available concerning the predictive value of egfr protein expression. In both the br.21 (*n* = 142: 11% vs. 4%, *p*= 0.1)^85^ and isel trials (*n* = 303: 8.2% vs. 3.2%)^83^, higher response rates to erlotinib were demonstrated for patients with egfr expression. However, the presence of *KRAS* mutations appears to be associated with a lower chance of tumour response. Lower response rates were observed in the br.21 (*n* = 118: 5% vs. 10%, *p* = 0.069)^85^, isel (*n* = 93: 0% vs. 8%)^83^, and tribute trials (*n* = 264: 8% vs. 23%, *p* = 0.16)^82^,^84^, although none of those results was statistically significant.

###### Molecular Predictors of Survival

TABLE X summarizes trials examining molecular predictors of survival for patients treated with an egfr-tki 71,74,75,77,80,82–86. No single molecular marker has consistently been associated with improved survival for patients treated with an egfr-tki.

The ideal 1 and 2 trials, br.21, and isel all examined single-agent egfr-tkis 71,77,83,85. Analysis of tumour samples from ideal 1 and 2 showed no significant improvement in ttp or survival for patients with *EGFR* mutations or with *EGFR* amplification^80^. However, these trials were not designed to examine predictors of survival, given that both groups of patients received an egfr-tki 80.

The br.21 trial generated several reports of molecular analyses^71^,^77^,^85^. On univariate analyses, there was no evidence that the survival benefit of erlotinib was influenced significantly by egfr expression (*n* = 325: ihc + hr: 0.68; ihc^−^ hr: 0.93; *p* = 0.1), increased *EGFR* gene copy (*n* = 159: fish^+^ hr: 0.43; fish^−^ hr: 0.80; interaction *p* = 0.12), or *EGFR* mutation status (*n* = 204: mut^+^ hr: 0.55; mut^−^ hr: 0.74; interaction *p* = 0.47). However, in multivariate analysis, increased *EGFR* gene copy was prognostic for poorer survival (*p* = 0.0025) and predictive of a differential survival benefit from erlotinib (*p* = 0.005)^71^,^77^,^85^.

The molecular analysis of the isel trial demonstrated a differential effect of gefitinib on survival according to *EGFR* gene copy (*n* = 370: fish + hr 0.61 vs. fish^−^ hr 1.16; interaction *p* = 0.045) and egfr expression (*n* = 379: ihc + hr: 0.77; ihc^−^ hr: 1.57; interaction *p* = 0.049). The data were insufficient for a survival analysis for patients with and without *EGFR* mutations^83^.

Molecular analyses are available from all four trials evaluating the addition of an egfr-tki to platinum-based chemotherapy. The addition of gefitinib to chemotherapy did not significantly improve os in patients with (hr: 2.03; 95% ci: 0.67 to 6.13) or without (hr: 1.01; 95% ci: 0.79 to 1.29) *EGFR* amplification (*n* = 453), or with (hr: 1.77; 95% ci: 0.5 to 6.2) and without (hr: 0.91; 95% ci: 0.67 to 1.23) *EGFR* mutations (*n* = 312)^86^.

Survival analysis from the tribute trial demonstrated a borderline improvement in ttp for patients receiving chemotherapy plus erlotinib (ttp hr: 0.59; 95% ci: 0.35 to 0.99), but no difference in os for patients with *EGFR* amplification (*n* = 245) 82,84. In patients with an *EGFR* mutation, there was also a trend toward improved ttp (12.5 months vs. 6.6 months, *p* = 0.092), but no difference in os was demonstrated (*p* = 0.96, *n* = 274)^82^,^83^. Similar findings were observed in the talent study. The presence of *EGFR* mutations did not predict for improved os (*p* = 0.65 for placebo vs. *p* = 0.40 for erlotinib) and pfs (*p* = 0.74 for placebo vs. *p* = 0.18 for erlotinib) irrespective of treatment^74^,^75^.

Information is more consistent that the presence of *KRAS* mutations is associated with worse survival for patients receiving an egfr-tki. Results from br.21 demonstrated a trend towards worse survival for patients on erlotinib with *KRAS* mutations (*n* = 206: *KRAS*^+^ hr: 1.67; *KRAS*^−^ hr: 0.69; *p* = 0.09) 71,77,85. Similarly, *KRAS* mutations predicted poor overall survival in erlotinib-treated patients on the talent trial 74,75. In addition, data from the tribute trial demonstrated that the presence of *KRAS* mutations was associated with significantly decreased ttp and survival in patients randomized to erlotinib plus chemotherapy (*n* = 274: hr: 2.1; 95% ci: 1.1 to 3.8; 4.4 months vs. 13.5 months *KRAS*^+^ vs. 12.1 months vs. 11.3 months *KRAS*^−^, *p* = 0.019]^82^,^84^.

In contrast, there is no evidence that these molecular markers predict a differential effect on survival from an egfr-tki than from chemotherapy. The molecular analyses from the interest trial showed no significant differences in survival between patients treated with gefitinib or with docetaxel according to egfr expression, *EGFR* gene copy, *EGFR* mutational status, or *KRAS* status^79^.

##### Consensus Recommendation

Molecular markers such as *EGFR* high gene copy or *EGFR* mutations and clinical characteristics such as adenocarcinoma, female sex, never smoking, and Asian ethnicity appear to be associated with a higher likelihood of response to an egfr-tki. The evidence is currently insufficient to select patients based on molecular markers predictive of improved survival with an egfr-tki. Prospective data will be needed before further recommendations can be made.

The evidence is conflicting about the predictive value of clinical characteristics for survival. However, the data suggest that the survival benefit of an egfr-tki may be greater among never smokers. Based on available data, molecular markers and clinical characteristics should not be used to exclude patients from receiving egfr-tki therapy.

## 4. DISCUSSION

The egfr-tkis of represent a significant advance in the management of advanced and metastatic nsclc. Not only do they have activity in nsclc, they also appear to have an improved toxicity profile as compared with standard chemotherapy agents such as docetaxel. As a result, they offer an attractive therapeutic option. Nevertheless, it is important that these agents be incorporated into routine treatment algorithms based on appropriate data from randomized trials.

It is clear that egfr-tkis should not be used concomitantly with standard chemotherapy agents in the treatment of nsclc. The strongest evidence supporting their use is in patients who have progressed following platinum-based chemotherapy. It is appealing to think that use of an egfr-tki may spare patients the toxicity of more chemotherapy. However, available data support the use of second-line chemotherapy and third-line egfr-tki or second-line egfr-tki and then third-line chemotherapy. Because both approaches prolong survival, the goal of therapy in advanced nsclc should be to maximize the number of patients who receive three lines of therapy, if survival is the outcome of interest. However, some patients will choose not to have second-line chemotherapy, and so the sequence of therapies should reflect a discussion between the physician and the patient regarding the relative benefits and side effects of each treatment option.

Multiple reports in the literature suggest that molecular markers and clinical characteristics can be used to select patients who will be more likely to benefit from an egfr-tki. However, this literature comes with significant limitations. The term “benefit” creates confusion, because it is used to refer to a variety of outcomes, including tumour response, improved os, and improved symptom control and qol. The molecular analyses are limited to patients whose tumour samples were available. The percentage of patients whose samples were available for one or more molecular analyses ranged from 25% to 44% of the total study population. As a result, some of these comparisons involve small numbers of patients. In addition, much of the literature has focused on tumour response rates, rather than on survival. Although there is some consistency in factors predicting response, these factors do not correlate directly with variables predicting a **differential** benefit in survival. Considerable variation is found in the variables reported to be associated with a differential improvement in survival from therapy with an egfr-tki. This variation may exist in part because some of the egfr markers are prognostic and associated with trends toward better survival (some *EGFR* mutations) or worse survival (high *EGFR* copy number). Therefore, it is not possible to assess the effect of egfr-tki therapy on survival in the absence of a no-treatment control arm. Furthermore, markers that seem to predict for a differential survival benefit when egfr-tki therapy is compared with placebo or no treatment may not be predictive when egfr-tki therapy is compared with another form of treatment such as chemotherapy. As a result, the evidence is currently insufficient to recommend the routine use of molecular markers and clinical characteristics to select patients for therapy with an egfr-tki. It is therefore also premature to recommend the use of single-agent egfr-tkis as first-line therapy for nsclc, even in patients selected on basis of molecular and clinical characteristics.

These results highlight the need for prospective trials in which tumour samples are available for all patients, so as to address correlative questions. Ongoing research will also address questions concerning the sequence of platinum-based chemotherapy or egfr-tki as first-line therapy.

Since the literature search for the present review was completed, preliminary data from two trials of maintenance gefitinib or erlotinib in Asian populations were presented at the American Society of Clinical Oncology Annual Scientific Meeting in 2008^87^,^88^. Both trials showed improved pfs, but no significant improvements in os. In addition, initial results of ipass (Iressa Pan ASia Study) were presented at the 2008 meeting of the European Society for Medical Oncology^89^. That trial compared first-line gefitinib with carboplatin and paclitaxel in light- or never-smoking Asian patients. A significant improvement was observed in pfs, but no significant difference in os. Other ongoing trials are evaluating the role of an egfr-tki as maintenance therapy in patients responding to first-line platinum-based chemotherapy.

Lastly, chemotherapy experience suggests that the therapeutic ratio can be improved with combination therapy. Preliminary evidence suggests that combination therapy with an egfr-tki and agents active against vascular endothelial growth factor may have greater activity. These questions are being addressed in multiple ongoing clinical trials. Participation in these trials should be encouraged.

## Figures and Tables

**TABLE I:** Trials of single-agent epidermal growth factor receptor tyrosine kinase inhibitors (egfr-tkis) in chemonaïve patients with non-small-cell lung cancer

									Survival	
Reference	Design	Treatment (daily dose)	Population	Patients (*n*)	Stage iii/iv (%)	ps 0–1/2 (%)	Response rate or/sd/pd (%)	ttp or pfs	Median	1-Year (%)
Pérez–Soler *et al.*, 2004^17^	Phase ii	Erlotinib 150 mg	Unselected	57	15.8/84.2	87.7/12.3	12^a^/35/49	9 Weeks	8.4 Months^a^	40
Kasahara *et al.*, 2005^18^	Phase ii	Gefitinib 250 mg	Unselected Asian	30	nr	nr	33/30	3.3 Months	10 Months	43.3
Spigel *et al.*, 2005^19^	Phase ii	Gefitinib 250 mg	Unselected	72	nr	0/83	4^b^/46/26	3.7 Months	6.3 Months	24
Swinson *et al.*, 2005^20^	Phase ii	Gefitinib 250 mg	Unselected	45	nr	17/27	9.8/36.6/53.4	32 Days	82 Days	nr
Akerley, 2006 21 and\Akerley et al., 2006^22^	Phase ii	Erlotinib 150 mg	Unselected	40	nr	100/0	15^b^/28/58^c^	22 Weeks	49 Weeks^c^	49^c^
Asahina *et al.*, 2006^23^	Phase ii	Gefitinib 250 mg	Selected (egfr^+^)^d^ Asian	16	nr	nr	75/6/19	8.9 Months	Not reached	Not reached
Giaccone *et al.*, 2006^24^	Phase ii	Erlotinib 150 mg	Unselected	53	21/79	85/15	23/30/23 (84 days)	3.0 Months (391 Days)	13.9 Months	54
Inoue *et al.*, 2006^25^	Phase ii	Gefitinib 250 mg	Selected (egfr^+^)^d^ Asian	16	0/63	88/12	75^b^/12.5/12.5	9.7 Months	nr	nr
Lin *et al.*, 2006^26^	Phase ii	Gefitinib 250 mg	Unselected Asian	53	13/87	76/9	32^b^/21/47	3.2 Months	9.4 Months	41.5
Niho *et al.*, 2006^27^	Phase ii	Gefitinib 250 mg	Unselected Asian	42	8/85	100/0	30^b^/40/30	nr	13.9 Months	55
Paz–Ares *et al.*, 2006^28^,^29^	Phase ii	Erlotinib 150 mg	Selected (egfr^+^)^d^	37	10/90	33/55	90/5/5^c^	13.3 Months	nr	82
Reck *et al.*, 2006^30^	Phase ii	Gefitinib 250 mg	Unselected	58	nr	76/24	5/40/52	7 Weeks	29 Weeks	
Suzuki *et al.*, 2006 31	Phase ii	Gefitinib 250 mg	Unselected Asian	34	0/100	100/0	26.5/23.5	nr	14.1 Months	58.2
Yang *et al.*, 2006 32	Phase ii	Gefitinib 250 mg	Unselected Asian	44	nr	40/4	54.5/20	6.3 Months	nr	n
Goss *et al.*, 2007 33	Randomized phase ii	Gefitinib 250 mg Placebo	Unselected	100 101	nr	0/100	6.0/–/not available 1.0/–/not available *p*=ns *p*=0.217	hr: 0.82 95% ci: 0.60 to 1.12 *p*=0.272	hr: 0.84 95% ci: 0.62 to 1.15	
Hesketh *et al.*, 2007^34^,^35^ (swog S0341)	Phase ii	Erlotinib 150 mg	Unselected	82	12/88	0/100	7^b^/36/42^c^	2 Months	6 Months	24
Jackman *et al.*, 2007^36^	Phase ii	Erlotinib 150 mg	Unselected, ≥70 years of age	80	15/85	90/10	10/41/35	3.5 Months	10.9 Months	46
Jackman *et al.*, 2007^37^	Phase ii	Erlotinib 150 mg	Selected based on patient characteristics	40 (women)	nr	100/0	30^b^/28/25	5.6 Months	Not reached (exceeds 23 Months)	nr
Jimenez *et al.*, 2007^38^,^39^	Phase ii	Erlotinib 150 mg	Unselected	437	24/76	70/30	31	6.6 Months	7.1 Months	nr
Sugio *et al.*, 2007^40^	Phase ii	Gefitinib 250 mg	Selected (egfr^+^)^d^ Asian	16	nr	nr	50/33/not available	8.8 Months	15.4	

a Predictor of overall response (multivariate analysis): time from last chemotherapy (*p* = 0.033); predictors of survival (multivariate analysis): time from initial diagnosis (*p* = 0.0007); good performance status [PS 0–1/2 (*p* = 0.04)].

b Partial response.

c 14 Patients could not be evaluated, and 1 patient experienced early death.

d Selected based on presence of egfr mutations.

ps = performance status; or = overall response (complete response + partial response); sd = stable disease; pd = progressive disease; ttp = median time to progression; pfs = median progression-free survival; nr = not recorded; hr = hazard ratio; ci = confidence interval; swog = Southwest Oncology Group.

**TABLE II:** Trials involving patients with adenocarcinoma with features of bronchioloalveolar carcinoma (bac)

									Survival	
Reference	Design	Treatment (daily dose)	Population	Patients (*n*)	Stage iii/iv (%)	ps 0–1/2 (%)	Response rate or/sd/pd (%)	pfs or ttp	Median	1-Year (%)
Miller *et al.*, 2006^42^	Phase ii	Erlotinib 150 mg	bac	102	nr	nr	21^a^	3.7 Months	17.1	
West *et al.*, 2006^43^ (SO126)	Phase ii	Gefitinib 500 mg	Previously untreated	101	7/93	90/10	17/32/33	4 Months	13	
			Previously treated	35	6/94	86/14	9/36/36	3 Months	13	
Cadranel *et al.*, 2007^44^ and ifct^45^ (IFCT0401)	Phase ii	Gefitinib 250 mg	Adenocarcinoma, bac	88	0/100	82/18	13/16/13	2.9 Months^b^	13.2^b^	53.4

a Response rate.

b Shorter progression-free and overall survival were independently associated with non-mucinous as compared with mucinous bac (pfs: 11.3 months vs. 2.6 months; *p* = 0.002; os: not reached vs. 10.7 months; *p* = 0.003).

ps = performance status; or = overall response (complete response + partial response); sd = stable disease; pd = progressive disease; pfs = median progression-free survival; ttp = median time to progression; nr = not recorded; ifct = Intergroupe Francophone de Cancerologie Thoracique.

**TABLE III:** Randomized trials of first-line epidermal growth factor receptor tyrosine kinase inhibitors (egfr-tkis) in combination with platinum-based chemotherapy in patients with non-small-cell lung cancer

								Survival	
Reference	Study	Treatment	Patients (*n*)	Stage iii/iv (%)	ps 0–1/2 (%)	Response rate or/sd/pd (%)	ttp or pfs	Median	1-Year (%)
Giaccone *et al.*, 2004^46^	intact 1	Cis–gem + placebo	363	30/69	90/10	47.2	6.0 Months	10.9 Months	44
		Cis–gem + gefitinib 250	365	27/72	90/10	51.2	5.8 Months	9.9 Months	41
		Cis–gem + gefitinib 500	365	33/67	90/10	50.3 *p*=ns	5.5 Months *p*=0.763	9.9 Months *p*=0.456	43
Herbst *et al.*, 2004^47^	intact 2	Carbo–pac + placebo	345	21/78	91/9	28.7	5.0 Months	9.9 Months	42
		Carbo–pac + gefitinib 250	345	19/81	90/10	30.4	5.3 Months	9.8 Months	41
		Carbo–pac + gefitinib 500	347	18/82	87/13	30.0 *p*=ns	4.6 Months *p*=0.056	8.7 Months *p*=0.638	37
Herbst *et al.*, 2005^48^	tribute	Carbo–pac + placebo	533	18/82	99.8/0.2	19.3	4.9 Months	10.5 Months	43.8
		Carbo–pac + erlotinib 150	526	16/84	100/0	21.5 *p*=0.36	5.1 Months *p*=0.36	10.6 Months *p=*0.95	46.9
Gatzemeier *et al.*, 2007^49^		Cis–gem + placebo	579	33/67	99/<1	31.5	23.7 Weeks	43 Weeks	41
		Cis–gem + erlotinib 150	580	35/65	100/<1	29.9 *p*=0.74	24.6 Weeks *p*=0.49	44.1 Weeks	42

ps = performance status; or = overall response (complete response + partial response); sd = stable disease; pd = progressive disease; ttp = median time to progression; pfs = median progression-free survival; intact = Iressa nsclc Trial Assessing Combination Treatment; Cis–gem = gemcitabine 1250 mg/m^2^ intravenously on days 1 and 8, and cisplatin 80 mg/m^2^ intravenously on day 1 of a 21-day cycle; erlotinib 150 = erlotinib 150 mg daily; gefitinib 250 = gefitinib 250 mg daily; gefitinib 500 = gefitinib 500 mg daily; ns = statistically nonsignificant; Carbo–pac = carboplatin auc (area under the curve) 6 intravenously on day 1, and paclitaxel 200 mg/m^2^ intravenously on day 1 of a 21-day cycle; tribute = Tarceva Responses in Conjunction with Paclitaxel and Carboplatin.

**TABLE IV:** Randomized trials of single-agent epidermal growth factor receptor tyrosine kinase inhibitors (egfr-tkis) compared with chemotherapy in chemonaïve patients with non-small-cell lung cancer

								Survival	
Reference	Design	Treatment	Patients (*n*)	Stage iii/iv (%)	ps 0–1/2 (%)	Response rate or/sd/pd (%)	ttp or pfs	Median (months)	1-Year
Crinò *et al.*, 2007^50^, ^a^ (invite)	Phase ii	Gefitinib	97	20/80	76/24	3.1/40	hr: 1.19		
		Vinorelbine	99	26/74	83/16	5.1/48	95% ci: 0.85 to 1.65 *p*=0.310		
Riely *et al.*, 2007^51^, ^b^	Phase ii	Erlotinib 150 mg + carboplatin/paclitaxel	87	nr	nr	18		12	
		Erlotinib 1500 mg + carboplatin/paclitaxel				35		16	
		Carboplatin/paclitaxel + erlotinib 1500 mg				24		nr (>9 )	
Lilenbaum *et al.*, 2008^52^, ^v^	Phase ii	Erlotinib	52	13/87	0/100	2/37/44	1.9 months	6.6 c	nr
		Carboplatin + paclitaxel	51	14/86	0/100	12/41/20	3.5 months	9.1 c	

a Gefitinib 250 mg daily compared with vinorelbine 30 mg/m^2^ intravenously on days 1 and 8 in a 21-day cycle.

b Erlotinib 150 mg on days 1 and 2, and carboplatin [area under the curve (auc) 6] and paclitaxel (200 mg/m^2^) on day 3; erlotinib 1500 mg on days 1 and 2, and carboplatin (auc 6) and paclitaxel (200 mg/m^2^) on day 3; or carboplatin (auc 6) and paclitaxel (200 mg/m^2^) on day 1 and erlotinib 1500 mg on days 2 and3. Patients received up to six 21-day cycles of treatment.

c Erlotinib 150 mg daily compared with carboplatin–paclitaxel [area under the curve (auc) 6 and 200 mg/m^2^ respectively) for 4 cycles.

ps = performance status; or = overall response (complete response + partial response); sd = stable disease; pd = progressive disease; ttp = median time to progression; pfs = median progression-free survival; nr = not recorded; invite = Iressa in nsclc vs Vinorelbine Investigation in the Elderly; hr = hazard ratio; ci = confidence interval.

**TABLE V:** Randomized trials of epidermal growth factor receptor tyrosine kinase inhibitors (egfr-tkis) as second- or third line therapy following progression of platinum-based chemotherapy

				Treatment line								Survival		
Reference	Design	Treatment	Pts (n)	2 (%)	3+ (%)	Prior platinum/taxane (%)	pd with prior chemotherapy (%)	Stage iii/iv (%)	ps 0–1/2 (%)	Response rate or/sd/pd (%)	ttp or pfs (months)	Median (months)	1-Year (%)	os*p* value
Fukuoka *et al.*, 2003 54, a (ideal 1)	Phase ii	Gefitinib 250	104	66	44	100/nr	nr	22/88	88/12	18.4/36/41	2.7	7.6	35	>0.05
		Gefitinib 500	106	67	33	100		17/83	87/13	19.0/32/42 *p*=ns	2.8	8.0	29	
Kris *et al.*, 2003 55, b (ideal 2)	Phase ii	Gefitinib 250	102	0	40/58	100/nr	nr	15/85	81/19	12/–/–	nr	7.0	27	0.54
		Gefitinib 500	114	0	42/58	100		8/92	79/20	9/–/– *p*=0.51		6.0 *p*=0.40	24 *p*=0.54	
Shepherd *et al.*, 2005 56, c (br.21)	Phase iii	Erlotinib 150	488	51	49	92/nr	28	nr	66/34^d^	8.9/36/45	2.2	6.7	31	<0.001
		Placebo	243	50	50	91.8/nr	28		68/32^d^	<1/27/57 *p<*0.001	1.8	4.7	21	
Thatcher *et al.*, 2005 57, e (isel)	Phase iii	Gefitinib 250	1129	49	50/1	96/27	38	44/47	65/29	8/32/37	3.0	5.6	27	0.087
		Placebo	563	49	50/1	96/28	40	39/50	68/26	1/31/48 *p*<0.0001	2.6	5.1	21	

a Gefitinib 250 mg daily vs. gefitinib 500 mg daily.

b Gefitinib 250 mg daily vs. gefitinib 500 mg daily.

c Erlotinib 150 mg daily vs. placebo.

d Includes patients with performance status 3 (8.6% in each arm).

e Gefitinib 250 mg daily vs. placebo.

Pts = patients; pd = progressive disease; ps = performance status; or = overall response (complete response + partial response); sd = stable disease; ttp = median time to progression; pfs = median progression-free survival; os = overall survival; nr = not recorded; isel = Iressa Survival Evaluation in Lung Cancer; ideal = Iressa Dose Evaluation in Advanced Lung Cancer; ns = statistically nonsignificant.

**TABLE VI:** Randomized trials of epidermal growth factor receptor tyrosine kinase inhibitors (egfr-tkis) compared with chemotherapy following progression after platinum-based chemotherapy

				Treatment line								Survival	
Reference	Design	Treatment	Pts (*n*)	2 (%)	3+ (%)	Prior platinum/taxane (%)	pd with prior chemotherapy (%)	Stage iii/iv (%)	ps 0–1/2 (%)	Response rate or/sd/pd (%)	ttp or pfs	Median	1-Year (%)
Cufer *et al.,* 2006 59, a (sign)	Phase ii	Gefitinib 250	68	97	—	91/0	nr	nr	63/37	13.2	3.0 months	7.5 months	nr^b^
		Docetaxel 75	73	99	—	96/0			71/29	13.7	3.4 months *p*=0.88	7.1 months	
Natale *et al.*, 2006 60, c	Phase ii	ZD6474	83	100		100/–	100	nr	100/–	–/45/–	11 weeks	6.1	nr
		Gefitinib 250	85	100	—	100/–	100			–/34/–	8.1 weeks *p*=0.025	7.4	
Chen *et al.*, 2007 61, d	Phase ii	Gefitinib 250	27	—	100	100/–	nr	nr	59/37	55.6	7.1 months	13.3 months	51.3
		Gefitinib 250 + vinorelbine 15	21	—	100	100/–			76/24	52.4	12.8 months *p*=0.133^e^	23.4 months	75.3
Douillard *et al.*, 2007 62, f (interest)	Phase iii	Gefitinib 250	733	100	100/–	26	14/86	88/12	9.1	2.2 months	7.6 months	32	
		Docetaxel 75	733	100	100/–	25	13/87	88/12	7.6 *p*=0.3257	2.7 months	8.0 months	34	
Herbst *et al.*, 2007 63, g	Phase ii	Chemotherapy + placebo	41	100	—	100/–	36.6	nr	98/2	12.2/27	3.0 months	8.6 months	33.1
		Chemotherapy + bevacizumab	40	100	—	100/–	17.5		100/0	12.5/40	4.8 months	12.6 months	53.8
		Bevacizumab + erlotinib	39				33.3		100/0	17.9/33	4.4 months	13.7 months	57.4
Lynch *et al.*, 2007^64^, ^h^	Phase ii	Erlotinib 150	50 (total)	100	—	100	100	nr	100/–	17/–/–	2.7 months	nr	nr
		Erlotinib 150 + bortezomi								8/–/–	1.4 months		
Niho *et al.*, 2007 65, 66, i	Phase iii	Gefitinib 250	244	87	13	100/–	17	19/81	96/4	22.5/12/66	2.0 months	11.5 months	48
		Docetaxel 60	245	82	17	100/–	15	20/79	96/4	12.8/21/66	2.0 months	14.0 months *p*=0.33^e^	54

a Gefitinib 250 mg daily vs. docetaxel 75 mg/m^2^ intravenously every 3 weeks.

b 6-Month survival rates were 65.6% with gefitinib and 56.1% with docetaxel.

c ZD6474 300 mg daily vs. gefitinib 250 mg daily.

d Gefitinib 250 mg daily vs. vinorelbine 15 mg/m^2^ intravenously on day 1, and gefitinib 250 mg daily on days 2–14 every 2 weeks.

e Overall survival.

f Gefitinib 250 mg daily vs. docetaxel 75 mg/m^2^ intravenously every 3 weeks.

g Chemotherapy (docetaxel or pemetrexed); bevacizumab 15 mg daily intravenously on day 1 of each 3-week cycle (± 5 days); erlotinib 150 mg daily for up to 52 weeks; docetaxel over 60 minutes (± 10 minutes) 75 mg/m^2^ on day 1 of a 3-week cycle (± 5 days); pemetrexed over 10 minutes (± 5 minutes) 500 mg/m^2^ on day 1 of a 3-week cycle. ^h^ Erlotinib150 mg daily vs. erlotinib 150 mg daily + bortezomib 1.6 mg/m^2^ intravenously on days 1 and 8 of a 21-day cycle. The study was halted as required at the planned interim analysis because of insufficient clinical activity in the erlotinib + bortezomib arm.

i Gefitinib 250 mg daily vs. docetaxel 60 mg/m^2^ intravenously every 3 weeks.

Pts = Patients; pd = progressive disease; ps = performance status; or = overall response (complete response + partial response); sd = stable disease; ttp = median time to progression; pfs = median progression-free survival; sign = Second-Line Indication of Gefitinib in nsclc; nr = not recorded; interest = Iressa non-small-cell lung cancer trial evaluating response and survival against Taxotere.

**TABLE VII:** Trials of clinical characteristics that predict response from therapy with epidermal growth factor receptor tyrosine kinase inhibitors (egfr-tkis)

Reference	Study	Patients (*n*)	Treatment	Adenocarcinoma	Never smokers	Female sex	Asian ethnicity
Kris *et al.*, 2003 55	ideal 2	216	Gefitinib 250 mg daily vs. gefitinib 500 mg daily	13% vs. 4%		19% vs. 3%	
Shepherd *et al.*, 2005 56	br.21	731	Erlotinib 150 mg daily vs. placebo	*n*=427	*n*=427	*n*=427	*n*=427
Clark *et al.*, 2006 71, ^a^				13.9% vs. 4.1%	24.7% vs. 3.9%	14.4% vs. 6%	18.9% vs. 7.5%
Florescu *et al.*, 2006 72				*p<*0.001	*p<*0.001	*p=*0.006	*p*=0.002
Tsao *et al.*, 2006 73							
Thatcher *et al.*, 2005 57	isel	1439	Gefitinib 250 mg daily vs. placebo	*n*=1439	*n*=1439	*n*=1439	*n*=1439
				11.9% vs. 4.8%	18.1% vs. 5.3%	14.7% vs. 5.1%	12.4% vs. 7.5%

isel = Iressa Survival Evaluation in Lung Cancer; ideal = Iressa Dose Evaluation in Advanced Lung Cancer.

**TABLE VIII:** Trials of clinical characteristics that predict survival from therapy with epidermal growth factor receptor tyrosine kinase inhibitors (egfr-tkis)

Reference	Design	Patients (*n*)	Treatment	Adenocarcinoma (HR)	Never smokers (HR)	Female sex (HR)	Asian ethnicity (HR)
Gatzemeier *et al.*, 2005 74,75 (talent)	Phase iii	1159	Erlotinib 150 mg daily vs. chemotherapy plus erlotinib 150 mg daily		Never-smoker hr: 0.39 *p=*0.25 Former-smoker hr: 1.05 *p*=0.86		
Thatcher *et al.*, 2005 57 (isel)		1439	Gefitinib 250 mg daily vs. placebo	0.84	0.69 vs. 0.92		0.66 vs. 0.92
Chang *et al.*, 2006 76 (isel)		342	Gefitinib 250 mg daily vs. placebo (subset of Asian population)	0.66 vs. 0.86	0.37 vs. 0.85	0.46 vs. 0.80	All-Asian population
Clark *et al.*, 2006 71,77,^a,b^		731	Erlotinib 150 mg daily vs. placebo	0.7 vs. 0.8	0.4 vs. 0.9	0.8 vs. 0.8	0.6 vs. 0.8
Florescu *et al.*, 2006 72							
Tsao *et al.*, 2006 73							
Shepherd *et al.*, 2007 78 (br.21)							
Douillard *et al.*, 2007 79 (interest)	Phase iii	1466	Gefitinib 250 mg daily vs. docetaxel	*p>*0.05	*p>*0.05	*p>*0.05	*p>*0.05

hr = hazard ratio; isel = Iressa Survival Evaluation in Lung Cancer; talent = Tarceva Lung Cancer Investigation Trial; interest = IIressa non-small-cell lung cancer trial evaluating response and survival against Taxotere.

**TABLE IX:** Trials of molecular characteristics that predict response from therapy with epidermal growth factor receptor tyrosine kinase inhibitors (egfr-tkis)

Reference	Design	Patients (n)	Treatment	Protein expression (ihc)	EGFR High gene copy (amplification ± high polysomy)	Mutations	KRAS Mutations
Bell *et al.*, 2005 80 (ideal 1/ideal 2, intact 1/intact 2)	Phase ii/iii	425	Gefitinib monotherapy (250 mg vs. 500 mg daily)		*n*=90 qpcr^+^ 29% vs. 15% *p*=0.319	*n*=79 46% vs.10% *p*=0.005	
		2130	Chemotherapy + gefitinib (250 mg daily or 500 mg daily)		*n*=235 qpcr^+^ 56% vs. 53% *p=*ns	*n*=170 mut^+^ 72% vs. 55% *p=*ns	
Eberhard *et al.*, 2005 82	Phase iii	1079	Erlotinib 150 mg daily		*n*=245^a^	*n*=228	*n*=264
Gatzemeier *et al.*, 2005^74^^,^^75^ (talent)	Phase iii	500	Chemotherapy + erlotinib 150 mg daily	*n*=375 No difference		*n* =293 *p*=ns	*n*=293 *p*=ns
Hirsch *et al.*, 2006 83 (isel)	Phase iii	1692	Gefitinib 250 mg daily + carboplatin/paclitaxel	*n*=303 ihc^+^ 8.2% vs. 1.5%	*n*=317 fish^+^ 16.4% vs. 3.2%	*n*=215 mut^+^ 37.5% vs. 2.6%	*n*=93 ras^+^ 0% vs. 8%
Hirsch *et al.*, 2007 84 (tribute)					fish^+^ 12% vs 22% *p*=ns	mut^+^ 53% vs. 18% *p<*0.01	ras^+^ 8% vs. 26% *p*=0.16
Zhu *et al.*, 2008 85 (br.21)		731	Erlotinib 150 mg daily	*n*=325 11% vs. 4% *p*=0.1^b^	*n*=159 fish^+^ 21% vs. 5% *p*=0.02^b^	*n*=204 mut^+^ 27% vs. 7% *p*=0.035^b^	*n*=206 ras^+^ 5% vs. 10% *p*=0.69

a Decrease in response.

b Univariate analysis.

ihc = immunohistochemistry; ideal = Iressa Dose Evaluation in Advanced Lung Cancer; intact = Iressa nsclc Trial Assessing Combination Treatment; qpcr = amplification determined by an increase in gene copy by a factor of 4 or more, as assessed by quantitative polymerase chain reaction; mut^+^ = mutation present; ns = statistically nonsignificant; fish = fluorescence *in situ* hybridization showing amplification or high polysomy; tribute = Tarceva Responses in Conjunction with Paclitaxel and Carboplatin; talent = Tarceva Lung Cancer Investigation Trial; isel = Iressa Survival Evaluation in Lung Cancer.

**TABLE X:** Trials of molecular characteristics that predict survival for therapy with epidermal growth factor receptor tyrosine kinase inhibitors (egfr-tkis)

Reference	Design	Patients (n)	Treatment	Protein expression (ihc)	EGFR High gene copy (amplification ± high polysomy)	Mutations	KRAS Mutations
Bell 2005 80, a (ideal and intact)	ideal 1/ideal 2, phase ii/iii	425	Gefitinib monotherapy (250 mg and 500 mg daily)		*n*=90 No difference in survival No difference in survival	*n*=119^b^ ttp: 116 days vs. 57 days	
	intact 1/intact 2, phase ii/iii	2130	Chemotherapy vs. Chemotherapy + gefitinib (250 mg or 500 mg daily)		*n*=453 fish^+^hr: 2.03 95% ci: 0.67 to 6.13 fish^−^hr: 1.01 95% ci: 0.79 to 1.29 *p=*ns	*n*=312 mut^+^hr: 1.77 95% ci: 0.5 to 6.2 mut^−^hr: 0.91 95% ci: 0.67 to 1.23 *p=*ns	
Eberhard *et al.*, 2005 82, c Hirsch *et al.*, 84, d 2007 (tribute)	Phase iii	1079	Erlotinib 150 mg daily + carboplatin/paclitaxel vs. placebo + carboplatin/paclitaxel		*n*=245 fish No difference in survival ttp hr: 0.59 95% ci: 0.35 to 0.99 os similar in both treatment arms	*n*=274 mut^+^ttp: 12.5 months vs. 6.6 months *p*=0.092 No difference in os *p*=0.96	*n*=274 ras^+^hr: 2.1 95% ci: 1.1 vs. 3.8 4.4 months vs. 13.5 months *p*=0.019 ras^−^ 12.1 months vs. 11.3 months
Gatzemeier *et al.*, 2005^74^,^75^ (talent)	Phase iii	500	Erlotinib 150 mg daily vs. chemotherapy + erlotinib 150 mg daily	*n*=375 No difference		os: *p*=0.40 (erlotinib), *p*=0.65 (placebo) *n*=293 pfs: *p*=0.18 (erlotinib), *p*=0.74 (placebo) *n*=293	os: *p*=0.51 (erlotinib) *n*=293 pfs: *p*=0.77 (erlotinib), *p*=0.22 (placebo) *n*=293
Clark *et al.*, 2006 71,77,e Tsao *et al.*, 2006 73, e Zhu *et al.*, 2008^85^, ^e^ (br.21)		731	Erlotinib 150 mg daily vs. placebo	*n*=325 ihc^+^hr: 0.68 95% ci: 0.49 to 0.95 ihc^−^hr: 0.93 95% ci: 0.63 to 1.36 *p*=0.1	*n*=159 fish^+^hr: 0.43 95% ci: 0.23 to 0.78 fish^−^hr: 0.80 95% ci: 0.49 to 1.29 Interaction *p*=0.12	*n*=204 mut^+^hr: 0.55 95% ci: 0.25 to 1.19^e^ mut^−^hr: 0.74 95% ci: 0.52 to 1.05 *p*=0.47	*n*=206 ras^+^hr: 1.67 95% ci: 0.62 to 4.50 ras^−^hr: 0.69 95% ci: 0.49 to 0.97 *p*=0.09
Hirsch *et al.*, 2006^83^ (isel)	Phase iii	1692	Gefitinib 250 mg daily and placebo	*n=*379 ihc^+^hr: 0.77 95% ci: 0.56 to 1.08 ihc^−^hr: 1.57 95% ci: 0.86 to 2.87 Interaction *p=*0.049	*n*=370 fish^+^hr: 0.61 95% ci: 0.36 to 1.04 fish^−^hr: 1.16 95% ci: 0.81 to 1.64 Interaction *p=*0.045		
Douillard *et al.*, 2007^79^ (interest)	Phase iii	1466	Gefitinib 250 mg daily vs. docetaxel	*p*=ns	*p*=ns	*p*=ns	*p*=ns
Garassino *et al.*, 2007^86^ (Pooled subset from isel, intact, tribute, and br.21)		1350	Gefitinib 250 mg daily vs. placebo Erlotinib 150 mg daily vs. placebo	*n*=325 egfr^+^hr: 0.72 egfr^−^hr: 1.08 Interaction *p*=0.048	*n*=578 fish^+^hr: 0.63 fish^−^hr: 1.03 Interaction *p*=0.022	*n*=447 mut^+^hr: 0.92 mut^−^hr: 0.85 Interaction *p*=0.796	

a intact 1: Chemotherapy (gemcitabine + cisplatin, *n*=363) + placebo vs. chemotherapy + gefitinib 250 mg daily (*n*=365) vs. chemotherapy + gefitinib 500 mg daily (*n*=365).

Gemcitabine 1250 mg/m^2^ intravenously on days 1 and 8; cisplatin 80 mg/m^2^ intravenously after gemcitabine on day 1 of a 21-day cycle; intact 2: Chemotherapy (paclitaxel + carboplatin) + placebo (*n*=345) vs. chemotherapy + gefitinib 250 mg daily vs. chemotherapy (*n*=345) + gefitinib 500 mg daily (*n*=347). Paclitaxel 225 mg/m^2^ intravenously on day 1; carboplatin [area under the curve (auc) 6] on day 1 of a 21-day cycle; ideal 1: gefitinib 250 mg daily (*n*=104) vs. gefitinib 500 mg daily (*n*=106); ideal 2, gefitinib 250 mg daily (*n*=102) vs. gefitinib 500 mg daily (*n*=114).

b Median time to progression: egfr mut^+^ > egfr mut^−^. No effect on overall survival.

c Univariate analysis.

d tribute: Chemotherapy (carboplatin + paclitaxel) + placebo vs. erlotinib 150 mg daily. Carboplatin [area under the curve (auc) 6] intravenously on day 1; paclitaxel 200 mg/m^2^ intravenously on day 1 of a 21-day cycle.

e tribute: Chemotherapy (carboplatin + paclitaxel) + placebo vs. erlotinib 150 mg daily. Carboplatin [area under the curve (auc) 6] intravenously on day 1; paclitaxel 200 mg/m^2^ intravenously on day 1 of a 21-day cycle.

ihc = immunohistochemistry; ideal = Iressa Dose Evaluation in Advanced Lung Cancer; intact = Iressa nsclc Trial Assessing Combination Treatment; ttp = time to progression; fish = fluorescence *in situ* hybridization showing amplification or high polysomy; hr = hazard ratio; mut+ = mutation present; ci = confidence interval; ns = statistically nonsignificant; tribute = Tarceva Responses in conjunction with Paclitaxel and Carboplatin; interest = Iressa non-small-cell lung cancer trial evaluating response and survival against Taxotere; isel = Iressa Survival Evaluation in Lung Cancer; talent = Tarceva Lung Cancer Investigation Trial; os = overall survival; pfs = median progression-free survival.
